# Awake, Arise, or Be for Ever Fall’n

**DOI:** 10.3201/eid1507.000000

**Published:** 2009-07

**Authors:** Polyxeni Potter

**Affiliations:** Centers for Disease Control and Prevention, Atlanta, Georgia, USA

**Keywords:** Art science connection, emerging infectious diseases, art and medicine, Pieter Bruegel the Elder, The Fall of the Rebel Angels, vector-borne diseases, Flemish painters, about the cover

**Figure:**
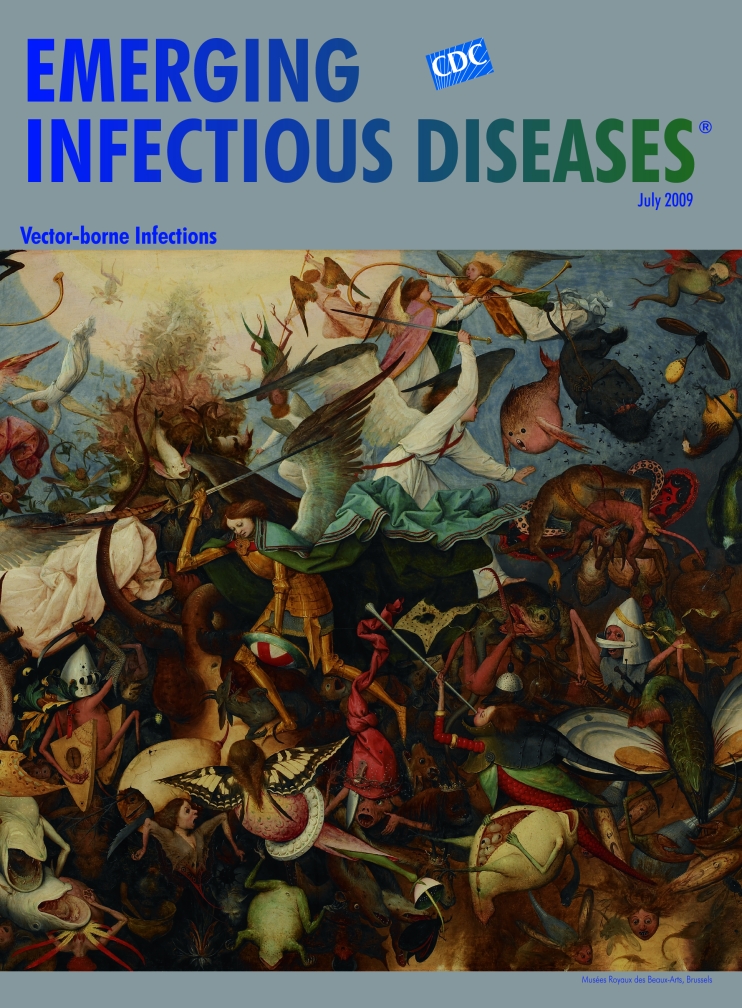
**Pieter Bruegel the Elder (c. 1525–1569) The Fall of the Rebel Angels (1562)** Oil on panel (117 cm × 162 cm) Musées Royaux des Beaux-Arts de Belgique, Brussels. Photo: Lefevre

―John Milton, Paradise Lost

“Pieter Bruegel was the most perfect of his century,” said Flemish cartographer and geographer Abraham Ortelius eulogizing his friend, who “was taken from us while still in his full manhood.” Both men observed and delineated new and original angles of reality, Ortelius in his great atlas (Theater of the World), Bruegel in his paintings, which described, in astonishingly modern terms, the lives of ordinary people. “When asked which of his predecessors he followed, the painter Eupompos is said to have declared that he followed nature herself, not an artist. This agrees with our Bruegel …. Indeed, I would not call him the best of painters, but rather the very nature of painters. So I think that he is worthy of being followed by all,” noted Ortelius, not alone in his praise.

“Nature was wonderfully felicitous in her choice when, in an obscure village in Brabant, she selected the gifted and witty Pieter Breughel to paint her and her peasants and to contribute to the everlasting fame of painting in the Netherlands,” wrote Karel van Mander in his Book of Painters in 1604. The obscure village was Breughel, a name the painter took for himself, though he later dropped the “h” in its spelling. Not much is known about him outside van Mander’s account. “He learned his craft from Pieter Koecke van Aelst, whose daughter he later married.” He settled in Antwerp and joined the guild of painters. He did much work for Hans Franckert, a merchant, who joined him often in his excursions among the peasants to know and paint them. He traveled to Rome but lived most of his 44 years in Antwerp and Brussels.

He was a “quiet and able man who did not talk much but was jovial in company, and he loved to frighten people, often his own pupils, with all kinds of ghostly sounds and pranks that he played.” His penchant for levity carried into his work. “He practiced a good deal in the manner of Jeroon [Hieronymus] van den Bosch and made many similar weird scenes and drolleries,” van Mander wrote, “Indeed, there are very few works from his hand that the beholder can look at seriously, without laughing. However stiff, serious, and morose one may be, one cannot help laughing or smiling.”

Mirth was welcome in Bruegel’s times, much as today. The Netherlands, then a kingdom under Spanish rule, was torn by religious fanaticism, and many were killed for heresy. Some of Bruegel’s works are allegories of the struggles between religious factions, but “it would be very hard to enumerate everything Bruegel did―fantasies, representations of hell, peasant scenes, and many other things …. He painted a picture in which Lent and Carnival are fighting; another, where all kinds of remedies are used against death; and one with all kinds of children at games; and innumerable other little, clever things.” His paintings were popular in his lifetime. They were collected by the Habsburgs and brought high prices; 49 works survive. Yet his reputation among the critics suffered from his overstated connection with Bosch and from his reluctance to follow the idealized styles prescribed by the Italian Renaissance.

Bruegel painted The Fall of the Rebel Angels, on this month’s cover, while he lived in Antwerp. This work, one of very few in the tradition of Bosch, shows Bruegel’s inclination toward the monumental, an aspect of the Italian Renaissance he did adopt. This, like many of his other works, shows a panoramic scene entirely filled with figures. The simplicity of form that characterizes these figures is what distinguishes Bruegel from other Flemish artists of his generation. The flat shapes, clean contours, and expressive faces devoid of extraneous detail are readily recognizable.

The fall of the rebel angels was described in the Book of Revelation as conflict between Archangel Michael and the forces of good against “that ancient serpent” and his fallen angels. The conflict, a favored theme in art from the Middle Ages onward, was commemorated in literary works from John Milton to Jonathan Edwards and William Blake. Bruegel approached the subject with his usual good humor, creating as a result a new version of Pandemonium. And moving away from biblical judgments, he fashioned for the good angels to remedy not so much a theological crisis as a “fine kettle of fish.”

While Bosch may be credited for inspiring this venture into the fantastic, Bruegel moved away from exotic ethereal creatures in favor of more earthy beasts with facial expressions, peering eyes, human limbs. These are no phantoms glowing in the painter’s imagination. They are pests―annoying, grotesque and interfering, prickly, buzzing, mocking, biting, threatening―even as they fall in total disarray into the depths of darkness. To make the scene more intriguing, Bruegel throws in realistically drawn detail: butterfly wings, an embellished robe, a grinning possum, a musical instrument or two, giving the scene more credibility and the fallen a certain wicked charm.

Fierce creatures as a metaphor for evil abound in literature. “Tyger! Tyger! Burning bright / in the forests of the night,” wrote William Blake (1757–1827) in Songs of Experience, “What immortal hand or eye / could frame thy fearful symmetry?” These famous lines generated countless discussions and interpretations, many about the possible link between Blake’s legendary carnivore and the struggle between good and evil in the world, between the tiger and “man in his fallen state.” “When the stars threw down their spears, / And watered heaven with their tears, / Did he smile his work to see? / Did he who made the lamb make thee?”

Lit by heaven, angels in flowing robes with the archangel in the lead, his princely gown billowing, are swatting dozens of monsters, while still more are hatching. Though outnumbered, the angels seem unfazed. The proceedings provide a tantalizing spectacle, not just of failed angels but of all monsters, among them nature’s ferocious bloodsucking nation of fleas, ticks, mosquitoes, and other horrors, pestering without provocation and spreading pain and disease, from epidemic typhus to bartonellosis, from rickettsioses to relapsing fever.

“I dried my tears and armed my fears / with ten thousand shields and spears,” wrote Blake in Songs of Experience, lamenting the loss of a guardian angel and building up personal defenses and strength. When it comes to swatting monsters in today’s infectious Bedlam, we can do no less. Like Bruegel’s angels we are outnumbered. Either we remain vigilant and keep the pests at bay, or we join them and fall.
